# Small-Bowel Capsule Endoscopy—Optimizing Capsule Endoscopy in Clinical Practice

**DOI:** 10.3390/diagnostics11112139

**Published:** 2021-11-18

**Authors:** Fintan O’Hara, Deirdre McNamara

**Affiliations:** 1 Department of Gastroenterology, Tallaght University Hospital, D24 NR0A Dublin, Ireland; mcnamad@tcd.ie; 2 TAGG Research Centre, School of Medicine, Trinity College, D24 NR0A Dublin, Ireland

**Keywords:** small bowel, capsule, endoscopy

## Abstract

The small bowel is the longest organ within the gastrointestinal tract. The emergence of small bowel capsule endoscopy (SBCE) over the last 20 years has revolutionized the investigation and diagnosis of small bowel pathology. Its utility as a non-invasive and well-tolerated procedure, which can be performed in an outpatient setting, has made it a valuable diagnostic tool. The indications for SBCE include obscure gastrointestinal bleeding, small bowel Crohn’s disease, and, less frequently for screening in polyposis syndromes, celiac disease, or other small bowel pathology. Currently, there are several small bowel capsules on the market from different manufacturers; however, they share many technological features. The European Society of Gastrointestinal Endoscopy (ESGE) only recently developed a set of key quality indicators to guide quality standards in this area. Many of the technical aspects of capsule endoscopy still feature a degree of uncertainty in terms of optimal performance. Incomplete studies due to slow transit through the bowel, poor imaging secondary to poor preparation, and the risk of capsule retention remain frustrations in its clinical utility. Capsule review is a time-consuming process; however, artificial intelligence and machine learning offer opportunities to improve this. This narrative review examines our current standing in a number of these aspects and the potential to further the application of SBCE in order to maximize its diagnostic utility.

## 1. Introduction

The small bowel is the longest organ within the gastrointestinal tract. Until the turn of the century, it was an obscure area in terms of endoscopic imaging, limited by the distance to which enteroscopes could be pushed into the small bowel. The emergence of small bowel capsule endoscopy (SBCE) has since revolutionized the investigation and diagnosis of small bowel pathology [1]. Its utility as a non-invasive and well-tolerated procedure, which can be performed in an outpatient setting, has made it a valuable tool for the gastroenterologist. Indications for SBCE include obscure gastrointestinal bleeding, small bowel Crohn’s disease (CD), and, less frequently for screening in polyposis syndromes, celiac disease, or other small bowel pathologies [2]. Currently, there are several small bowel capsules on the market, from different manufacturers; however they share many technological features, and all remain solely an imaging modality at present. The European Society of Gastrointestinal Endoscopy (ESGE) only recently developed a set of key quality indicators to guide quality standards in this area [3]. All areas related to capsule endoscopy still feature a degree of uncertainty as to how optimize performance. Incomplete studies due to slow transit through the bowel, poor imaging secondary to poor preparation, and the risk of capsule retention, continue to frustrate its clinical application. Another challenge faced in SBCE is the risk of missed pathology due to the subtle nature of lesions, as well as the often fleeting glimpses of the pathology obtained. This narrative review analyzes the current state of SBCE application and assesses potential opportunities to develop the application of SBCE in order to maximize its diagnostic utility.

## 2. Patient Selection/Timing

Pressure on diagnostic availability is a worldwide issue. Therefore, we must make sure that we maximize the diagnostic utility of this valuable resource through the careful selection of patients and optimal timing of the test.

### 2.1. Biomarkers

Biomarkers can assist us in helping to triage referrals to determine which patients are most likely to benefit from SBCE and to maximize diagnostic yield.

C-reactive protein (CRP) is a readily available biochemical marker of inflammation. The European Crohn’s and Colitis Organization recommends its usage in the diagnosis and monitoring of inflammatory bowel disease (IBD) [4]. A raised CRP broadly correlates with clinical severity in CD [5,6]. However, this has not been shown in small bowel capsule studies, where several studies have shown that it correlates poorly with positive findings on capsule endoscopy in the investigation of IBD. Kopylov et al. showed that significant small bowel inflammation correlates poorly with elevated CRP (r = 0.3; *p* = 0.01; sensitivity, 60.5%, specificity, 70.3%), elevated fecal calprotectin (ELISA > 200 µg/g, QB > 100 mg/g; r = 0.164, *p* = 0.07; sensitivity, 69.8%, specificity, 46.5%), or a combination of both elevated markers (r = 0.2; *p* = 0.14) [7].

Fecal calprotectin (FC) can also be used as a screening tool to help select patients referred with suspected small bowel inflammation. FC offers significant diagnostic accuracy for the detection of small-bowel CD [8]. In patients with suspected CD with an FC of less than 50 μg/g, the likelihood of finding significant small bowel inflammation is extremely low [9]. A good correlation has also been demonstrated between FC and the Lewis score on SBCE [10]. FC has also been shown to be a good predictor of the mucosal healing of CE in patients with established CD on treatment [10].

Using data from our group, Yousuf, et al. showed that the correlation between FC and SBCE results improved with increasing FC levels. Our study suggests that an FC level > 194 µg/g may be a useful filter test in patients referred for SBCE with either suspected or known small-bowel CD and could help to prioritize referrals. Depending on local capacity, selecting an FC cut-off of >100 or 200 µg/g could be effectively employed to triage more urgent procedures, as both feature a reasonable specificity of 75.5 and 89%, respectively. However, overall, the sensitivity for FC was felt to be inadequate and “clinical suspicion in the face of negative traditional endoscopy investigations should continue to trigger dedicated additional SB investigations.” [11]. Jung et al. showed that FC has significant diagnostic accuracy for detecting small bowel inflammation, and suggested that an FC cut-off of 100 µg/g can be used as a tool to screen for small bowel CD [8].

The fecal immunochemical test (FIT) is used widely in colorectal cancer screening. A 2017 small systematic review showed a low sensitivity and specificity of FIT for significant findings on CE where FIT used as a selection tool for SBCE showed a sensitivity of 0.48 and a specificity of 0.60; i.e., neither a positive nor a negative FIT were good predictors of SB findings on CE [12]. However, more recent data provided by our colleagues have suggested FIT to be a useful biomarker in predicting the likelihood of small bowel pathology in patients with anemia. Judge et al. found a statistically significant association between positive FIT (FIT >/= 45 μg Hb/g was considered positive) and pathology on SBCE (OR 12, 95% CI [2.8–51.9], *p* = 0.001). The sensitivity and specificity of positive FIT in predicting SBCE findings were 69 and 89%, respectively [13]. A negative FIT has also been shown to have a high negative predictive value for small-bowel lesions in this and other studies [13,14].

Thus far, FC seems to be a promising and cost-effective screening test prior to SBCE to identify patients with a higher likelihood of having small bowel inflammation, although there is no clear cut-off value at the time of writing. So far, FIT testing and CRP have not shown good clinical utility in this setting (Table 1).

### 2.2. Hemoglobin Levels

Iron-deficient anemia with negative upper and lower endoscopy forms a significant portion of referrals for capsule endoscopy. Olano et al., have demonstrated that, in patients with iron-deficient anemia who have been worked up with upper and lower endoscopy, capsule endoscopy is an efficient technique through which to find an underlying etiology. They showed in a multivariable analysis that low hemoglobin levels (OR 0.73; 95% CI 0.57–0.94) had an independent effect on the probability of obtaining positive findings in patients with IDA [15].

### 2.3. Timing

Emerging data on early SBCE in patients admitted with obscure gastrointestinal bleeding has shown that early procedures improve diagnostic yield. Marya et al., identified a bleeding source in 64.3% of patients receiving an early SBCE versus 31.1% of t patients receiving standard care (*p* < 0.01) [16]. Early SBCE also led to earlier endoscopic localization of bleeding compared with those in the standard of care arm (adjusted hazard ratio, 2.77; 95% confidence interval, 1.36–5.64) [16]. Others have also shown that earlier diagnosis of small bowel pathology can also lead to earlier intervention and a lower rebleeding rate [17]. There is a benefit to early SBCE, and this needs to be considered in the triage criterion used for an often oversubscribed service.

## 3. Procedural Issues

Small bowel cleanliness, along with obtaining a complete examination, are the key factors in the delivery of high diagnostic performance in SBCE. A total of 2 L of Polyethylene glycol (PEG)-based laxative is the most widely used and comes with an ESGE recommendation. Improvements in this regime and the question of whether they can improve diagnostic yield is still an area of discussion. Here, we assess recent advances in this area, as well as the usage of prokinetics to aid capsule transit.

### 3.1. Small Bowel Preparation

Small bowel preparation remains an area of much debate in SBCE. ESGE recommend a modified diet pre-procedure, as well as the ingestion of 2 L of a PEG-based purgative to improve visualization [18]. A number of meta-analyses have concluded that 2 L of PEG solution prior to capsule ingestion improves the visibility of the small bowel, but the question of whether this improves completion rates and diagnostic yield is still inconclusive, and the optimal timing of the purgative’s use is unclear [19,20]. The most recent published meta-analysis showed that in comparison with a clear-liquid diet, purgative bowel preparation did not increase the diagnostic yield (RR 1.17 (95% CI 0.97 to 1.40); *p* = 0.11), the quality of small-bowel mucosal visualization (RR 1.14 (95% CI 0.96 to 1.35); *p* = 0.15) nor the completion rate for the examination (RR 0.99 (95% CI 0.95 to 1.04); *p* = 0.76) [21].

### 3.2. Anti-Foaming Agents

ESGE recommends anti-foaming agents prior to capsule ingestion [18]. All three RCTs have demonstrated that anti-foaming agents improve the quality of mucosal visualization and two meta-analyses have concluded that simethicone significantly decreases the presence of small-bowel bubbles/foam [19,22]. There is no consensus on the optimal dose of simethicone, with ranges between 80 to 200 mg used [19]. A recent trial of high-dose simethicone (1125 mg in 750 mL of water) did not show any benefit for the high dose versus a standard dose of 300 mg [23].

### 3.3. Diving Method

Promising recent research published in GIE involves the ingestion of 500 mL of water every hour, administered after the capsule reached the small bowel, the so called “diving method” [24]. The scores for the endoscopic images in the proximal third and middle third of the small bowel in the “diving group” were significantly higher than those in the control group (3.47 ± 0.60 vs. 3.11 ± 0.63 (*p* = 0.007) and 3.24 ± 0.59 vs. 2.78 ± 0.74 (*p* = 0.002), respectively). The diagnostic yield in the distal third of the small bowel was significantly higher in the “diving group” (*p* = 0.005), as was overall completion rate (92.19% vs. 76.32%, respectively; *p* = 0.012). However, disappointingly, the lesion detection rates were similar in the two groups (*p* = 0.577). No adverse events were reported during the entire trial. No discomfort was recorded, except complaints of frequent urination from 5 patients out of 64 [24].

### 3.4. Prokinetics/Real Time Viewer

The major cause for incomplete examination of the small bowel is prolonged gastric transit time. Prokinetics have been used to aid the passage of the capsule from the stomach to the small bowel. However, prokinetic use alone has been shown to be ineffective at increasing SBCE completion rates [25]. However, patients with risk factors for an incomplete SBCE study (i.e., those with previous history of abdominal surgery, delayed gastric emptying, diabetic neuropathy, severe hypothyroidism, or use of psychotropic drugs) may benefit from the administration of certain prokinetics (metoclopramide, domperidone or erythromycin) when the capsule remains in the stomach for more than 30–60 min, as confirmed by real-time monitoring. The ESGE recommends prokinetic usage/or endoscopically assisted capsule delivery into the duodenum in patients for whom delayed gastric emptying has been confirmed by real time monitoring.

A prospective study [26] from Japan compared SBCE in 80 patients with or without real-time viewing. In this study, in the real-time viewing group, if the capsule remained in the stomach for more than 60 min, a prokinetic was administered, followed by PEG. The completion rate in the real-time viewing group was significantly higher than in the control group (90% vs. 72.5%). A further Japanese study [27] recently compared the proportion of completed exams and positive findings among a group of patients studied before the introduction of real-time viewing and a group in which capsule transit was regularly monitored and action was taken (e.g., the administration of water or intravenous metoclopramide) if it was delayed. The use of a real-time viewer increased the SBCE completion rates from 66% to 86% (*p* = 0.002). Our group has also demonstrated similar data, where the completion rates (90.2% (*n* = 48) vs. 96.25% (*n* = 102)) and the rates of significant findings (37.7% (*n* = 20) vs. 32.5% (*n* = 34)) were similar in our at-risk population who received prokinetics for delayed gastric emptying in comparison to a control group [28]. There were no serious adverse events in the prokinetic group, but four (7%) reported side effects including nausea, dizziness, and pain at the cannula site. A significant (15%) proportion required a second prokinetic, increasing the potential for adverse events, given that the use of metoclopramide and erythromycin as prokinetics often poses a significant risk of cardiac adverse effects in elderly cohorts. Alternative prokinetic interventions would be advantageous. Peppermint oil has been shown to function as a prokinetic when administered orally and provides a possible alternative to IV-administered medications [29].

Optimum bowel preparation with laxatives and other additives such as those discussed above remain a topic of debate within the study of capsule endoscopy. While a number of the studies mentioned above improved views and completion rates, this did not result in improvements in diagnostic yields. Whether it is possible to improve the standard PEG-based regime is still for a question for research and debate.

## 4. Capsule Retention

Capsule retention is defined as the identification of a capsule on abdominal radiological imaging ≥ 14 days after capsule ingestion, or the need for its surgical removal due to small bowel obstruction [30]. It remains a feared complication during capsule endoscopy. The identification of risk factors at patient triage and the use of the patency capsule have ameliorated this risk.

### 4.1. Risk Factors/Rates

We now possess a good understanding of the risk factors associated with capsule retention. The presence of a combination of symptoms suggestive of bowel obstruction (abdominal pain, abdominal distension, and nausea/vomiting) before capsule endoscopy has been shown to be associated with a significantly higher rate of capsule retention [31,32,33]. Furthermore, previous small-bowel resection, abdominal/pelvic radiation therapy, and the chronic use of high-dose nonsteroidal anti-inflammatory drugs (NSAIDs) have all been shown to increase the risk of capsule retention [31,32].

The most cited meta-analysis of capsule retention rates shows that retention occurs in approximately 2% of patients undergoing evaluation for small-bowel bleeding and between 4% and 8% in patients with suspected or known IBD [34]. This analysis overestimated the real-world retention rate in CD as it excluded studies that performed small bowel patency assessments routinely. Indeed, these rates decreased by half in those studies that used either a patency capsule or CT enterography to assess patency before performing VCE [34].

Indeed, Wang’s 2020 systematic review of 108,079 capsule endoscopy procedures showed a retention rate of 0.73% (Table 2). The retention rate (coefficient = −0.34%, 95% CI −0.53 to −0.14%, *p* = 0.0006) decreased significantly over the 20 years examined due to improved patient selection and the use of patency capsules. The incomplete examination rate of SBCE also significantly declined over time (*p* < 0.0001). As to the causes of capsule retention, CD was the most common retention reason (35.41%). Among the 766 retained capsules, surgery was the most frequently used intervention (*n* = 352, 45.95%), followed by endoscopic management (*n* = 199, 25.98%), no intervention (*n* = 176, 22.98%), and medical therapy (*n* = 39, 5.09%) [35].

When capsule retention does occur, it is usually asymptomatic [34], and the capsule can remain in the small bowel without symptoms for several months or be naturally egested during subsequent follow-up [36]. In a recent population study, only 1.9% out of 104 capsule retentions developed into a symptomatic bowel obstruction [36]. Thus, conservative observation is justified for the management of capsule retention in the majority of cases, unless malignancy is strongly suspected. During this period, targeted treatment with medications (including corticosteroids as appropriate), may promote capsule egestion in up to 20–30% of patients with capsule retention [reference]. The ESGE recommends observation in cases of asymptomatic capsule retention, and in cases where capsule retrieval is indicated, they recommend the use of device-assisted enteroscopy as the method of choice [18].

### 4.2. Patency Capsule

Patency capsule testing and dedicated small-bowel cross-sectional imaging techniques have both been found to be effective at assessing patency [37,38]. In a meta-analysis of patency capsule usage including five studies and 203 patients, the pooled data showed a patency capsule sensitivity of 97% (95% CI 93–99%) and a specificity of 83% (95% CI 65–94%) [39].

As stated previously, retention rates are falling. Compared with a 2010 study, Wang et al.’s 2020 systematic review detected a lower pooled retention rate of 0.73% vs. the 1.4% rate seen previously [32,35]. This could be attributed to the usage of patency capsules, which predicts small-bowel strictures in patients at high risk for retention. Wang’s results showed a retention rate after negative patency testing of 0.09% in a subgroup analysis, and patency testing demonstrated a significantly decreased retention rate by 5.04% in multivariate meta-regression [37]. These findings and others confirm that performing an initial patency capsule test before SBCE in patients with a high risk of retention is useful to avoid retention [35].

In studies published over the past five years, where assessment for patency was the standard procedure, retention rates in patients with established CD were much lower (up to 2.5%); indeed, in the largest study to date of 343 CE procedures performed in patients with established CD, a retention rate of 2.3% was observed [40].

The evaluation of small bowel (SB) patency in patients with established CD by means of a patency capsule or imaging is now the standard practice endorsed by the recent European Crohn’s and Colitis Organization/European Society of Gastrointestinal Endoscopy guidelines [18]. However, there is a risk of overuse of the patency capsule; indeed, ESGE does not recommend offering a patency capsule procedure indiscriminately to all patients undergoing capsule endoscopy. The rates of positive patency tests range from 30% [40] to 43% (our unpublished data). These rates are far higher than those seen in any real-world SBCE data. Thus, the overuse of patency capsules results in the follow-on exclusion of patients who may have benefited from the test. SBCEs after positive patency tests have been shown to have high retention rates: in 18 patients who underwent SBCE after a positive patency capsule test a retention rate of 11.1% was seen [40]. However, although this is a high result, the real capsule was still passed on 89% of positive patency tests. Are there ways of reducing the rates of “false positive” patency tests without affecting the retention rates? Extending the time for confirming functional patency up to 72 h only in cases in which an intact capsule or intact body of PPC is observed directly may be acceptable and may enhance the probability of performing CE safely. In 6 of 19 patients (31.6%) in whom patency was not confirmed at 30 h, patency was confirmed within 72 h and no capsule retention occurred [41].

In patients who have suspected CD, the risk of capsule retention is low, so using a patency capsule is not required routinely [18]. Nemeth’s 2015 study suggested that even in patients with known CD but no suspicion of strictures and/or abdominal complaints suggestive of small-bowel obstruction, there is no need to use a patency capsule [40]. A patency test is recommended, however, when the patient demonstrates previous occlusive symptoms, such as a combination of abdominal pain and distension, abdominal pain and nausea/vomiting, and abdominal distension and nausea/vomiting [36].

If a patient fails to notice the passage of the capsule within 30 h and subsequently undergoes a plain film X-ray, it can be difficult to accurately determine the location of the capsule because the images of the small bowel and colorectum can overlap. As a result, a subsequent fluoroscopy or CT scan may be needed in cases where the localization of the patency capsule is not definite. However, the availability of CT or fluoroscopy in this case is likely to be limited and, consequently, there is a risk of the patient being excluded from the test in error.

Great strides have been made in reducing the rate of capsule retention over the last twenty years (Table 3). Refining the use of the patency capsule with further study will hopefully help to reduce the exclusion of patients from capsule endoscopy unnecessarily.

## 5. Procedure Reporting/Artificial Intelligence-Who and How Should Capsules Be Read?

### 5.1. The Who

The reading of capsule studies is a time-consuming exercise, with a mean reading time of 45–60 min [42,43]. This raises the question as to who the most appropriate person is to read these studies. Previous evidence confirms that, with adequate training, nurses and/or other technical staff can identify pathology as well as physicians. The ESGE “recommends the acceptance of qualified nurses and trained technicians as prereaders of capsule endoscopy studies as their competency in identifying pathology is similar to that of medically qualified readers” with the important caveat that “the responsibility of establishing a diagnosis must remain with the treating physician” [18]. The ESGE have agreed on the suggestion that a minimum of 30–50 SBCEs is required to acquire proficiency and that 30–50 procedures per year are needed to maintain competence. The ESGE is also currently developing a curriculum for training in small-bowel endoscopy [3].

### 5.2. The How

The ESGE recommends a maximum reading speed of 10 frames per second (less in the proximal small bowel) for small-bowel capsules. Double- and multiple-view modes, if available, at a maximum speed of 20 frames per second have also been shown to be acceptable [18]. Imaging enhancing modes, collectively known as virtual chromoendoscopy, such as FICE “blue mode” and ALICE, have shown no benefit over white light imaging to date and are not recommended for routine usage [44,45].

### 5.3. The Future

Capsule endoscopy appears to be ideally suited to machine learning and artificial intelligence, as in its current guise it entails pattern recognition from still images. As technology improves, this offers the potential to speed up reading times by eliminating normal images, removing near-identical images, and, in the future, recognizing and identifying pathology. Indeed, in a recent meta-analysis that included 19 retrospective studies, deep learning applications for capsule endoscopy detection accuracy were above 90% for most studies and diseases [46]. The pooled sensitivity and specificity for ulcer detection were 0.95 (95% confidence interval [CI], 0.89–0.98) and 0.94 (95% CI, 0.90–0.96), respectively. The pooled sensitivity and specificity for bleeding or bleeding source were 0.98 (95% CI, 0.96–0.99) and 0.99 (95% CI, 0.97–0.99), respectively (Table 4). However, these retrospective studies featured an elevated risk of bias and further, larger-scale prospective studies are required to raise confidence in their clinical utility. Early prospective data point to comparable results in the real-world application of some of these technologies. AI mode showed a sensitivity, specificity, NPV and PPV of 94.6%, 100%, 83.3%, and 100% respectively in a 48 patient prospective study of a Mirocam Express view system published as an abstract. The mean reading time was 84.13 ± 53.63 min in the standard arm and 14.79 ± 11.2 min in the Express View arm (*p* < 0.001) [47].

The use of machine learning for the detection of multiple pathologies and lesion localization in SBCE is also under investigation, with continuing improvements refining the technology [48,49].

The assessment of bowel preparation quality is another area where computer algorithms are likely to be extremely useful. Currently, the main issue with the assessment of preparation quality and the various scores used is that they are operator-dependent and time-consuming [50]. Indeed, a universally accepted scale for grading small-bowel cleansing is still lacking. The available grading systems feature different technical characteristics, assessing different parameters and portions of the video. The ESGE does recommend that Brotz and Park scores should be used for SBCE [3].

To date, studies have used computer-generated scores using red-to-green pixel ratios that correlate well with operator-dependent scores. The concept of the R/G ratio is based on the principle that properly visible mucosa are associated with red colors, whereas a fecal-contaminated lumen is associated with green colors. In a study by Ali et al., their small bowel computer assessment of cleaning score demonstrated a sensitivity of 91.3% and a specificity of 94.7% in comparison to an experienced reader’s score assessing adequate small-bowel visualization [51].

More recently, Oumrani et al. proposed a score based on three electronic parameters: colorimetry, abundance of bubbles, and brightness. These parameters were compared to the Brotz score, as assessed by different experts, with a score of 7/10 representing adequate mucosal visualization. Through automated analysis, the combination of the R/G ratio, abundance of bubbles, and brightness achieved a sensitivity of 90.0% and a specificity of 87.7%, with optimal reproducibility. The limitations of this score analysis are that it was performed on still frames and not on video analysis and on normal videos of patients with obscure GI bleeding [52]. Although numerous automated scores have been proposed, to date, no practical and readily available score is available on the CE reading software, although such a score is highly likely to become available soon.

**Table 4:** Deep learning implementation for the detection of bleeding/bleeding source and ulcers in capsule endoscopy (data from Soffer et al. [45]).

Bleeding/Bleeding Source	Sensitivity (95% CI)	Specificity (95% CI)	Ulcer Detection	Sensitivity (95% CI)	Specificity (95% CI)
Aoki et al., 2019 [53]	0.99 (0.96–1.00)	1.00 (1.00–1.00)	Alasker et al., 2019 [54]	1.00 (0.95–1.00)	1.00 (0.86–1.00)
Jia et al., 2017 [55]	0.91 (0.84–0.96)	0.98 (0.97–0.99)	Aoki et al., 2019 [53]	0.87 (0.83–0.90)	0.91 (0.90–0.91)
jia et al., 2017 [56]	0.99 (0.98–1.00)	1.00 (0.99–1.00)	Fan et al., 2019 [57]	0.97 (0.95–0.98)	0.95 (0.94–0.96)
Leenhardt et al., 2019 [58]	1.00(0.99–1.00)	0.98 (0.93–0.98)	Klang et al., 2019 [59]	0.98 (0.97–0.98)	0.97 (0.97–0.97)
Tsuboi et al., 2019 [60]	0.99(0.97–1.00)	0.98 (0.98–0.99)	Wang et al., 2019 [61]	0.90 (0.89–0.91)	0.90 (0.89–0.91)
Pooled Estimate	0.98 (0.96–0.99)	0.99 (0.94–0.99)	Pooled Estimate	0.95 (0.89–0.98)	0.94 (0.90–0.96)

## 6. Conclusions

Small-bowel capsule endoscopy has revolutionized the investigation of small-bowel pathology over the last 20 years. There has never been a more exciting time in capsule endoscopy as developments in technology, including higher definition imaging, AI reading software, and extended battery life push the limits of the test. Even with the advances in technology, however, there is still much progress to be made on the numerous technical aspects of this procedure.

## Figures and Tables

**Table 1:** Calprotectin and FIT test sensitivity and specificity for significant findings on capsule endoscopy.

Test	Cut-Off			Sensitivity (95% CI)	Specificity (95% CI)	DOR (95% CI)	AUC
Faecal	>100 μg/g	Jung et al. (2017) [8]	14 studies (794 patients)	0.725 (0.66–0.78)	0.728 (0.62–0.81)	7.894 (4.32–14.44)	0.763
Calprotectin	>100 μg/g	Kopylov et al. (2016) [7]	7 studies (463 patients)	0.68	0.71	5.01	-
	>45 μg Hb/g	Judge et al. (2019) [13]	51 patients	0.692	0.889	-	0.84
FIT	>100 μg Hb/g	Yung et al. (2017) [12]	4 studies (478 patients)	0.48 (0.36–0.61)	0.60 (0.42–0.76)	1.41 (0.72–2.75)	-
	>100 μg Hb/g	Endo et al. (2017) [14]	157 patients	0.56 (0.35–0.75)	0.47 (0.44–0.49)	-	-

**Table 2:** Rates of capsule retention and trend over time (data from Wang et al. [35]).

(*n* = 108,079)	Rate	95% CI
Pooled Retention Rate	0.73%	0.59–0.89%
Established CD	4.29%	1.46–7.12%, (*p* = 0.0029)
Retention Post Patency Assessment	0.09%	0.00–0.34%

**Table 3:** Trends in retention rates (data from Wang et al. [35]).

(*n* = 108,079)	Co-Efficient	95% CI
Retention Reduction post Patency	−5.04%	−8.75% to −1.33%, (*p* = 0.0077)
Trend in Retention rates 2006–2016	−0.34%	−0.53 to −0.14%, (*p* = 0.0006)

## Data Availability

Not applicable.
